# Supplementary material for: Ecogeographical Adaptation Revisited: Morphological Variations in the Plateau Brown Frog along an Elevation Gradient on the Qinghai–Tibetan Plateau
Source: Biology (Basel). 2021 Oct 22;10(11):1081. doi: 10.3390/biology10111081 (PMC8614741; doi:10.3390/biology10111081)
Supplement: Supplementary file 1 [file biology-10-01081-s001.zip › biology-1397339-supplementary.pdf]

Table S1. Summary of location information and sample details from 10 surveyed populations.

Population ID	Location	Elevation (m)	Latitude	Longitude	Male				Female			
					SVL (mm)	LAHL (mm)	HILL (mm)	n	SVL (mm)	LAHL (mm)	HILL (mm)	n
1	Maoxiang'ping, Mao county	1797	31.71	103.91	43.53 ± 3.88	20.28 ± 1.68	72.56 ± 7.29	34	49.50 ± 5.42	21.37 ± 2.55	82.54 ± 10.02	3
2	Lianghe'kou, Mao county	2296	32.06	103.64	50.20 ± 5.83	23.33 ± 2.68	84.35 ± 11.42	17	63.77 ± 7.81	28.84 ± 4.84	104.22 ± 13.64	4
3	Baila'hai, Mao county	2526	32.08	103.64	46.69 ± 6.01	21.46 ± 2.60	76.64 ± 9.15	18	-	-	-	0
4	Yanwo'cun, Mao county	2954	32.18	103.49	53.38 ± 3.99	25.04 ± 2.61	88.81 ± 7.21	27	57.62 ± 7.77	25.90 ± 2.38	90.74 ± 9.12	5
5	Mouni'gou, Songpan county	2769	32.51	103.61	50.44 ± 6.21	24.24 ± 4.07	84.93 ± 12.03	6	49.93 ± 6.45	22.34 ± 2.05	81.56 ± 9.90	8
6	Chuanzhu'si, Songpan county	3022	32.74	103.60	51.85 ± 2.76	23.15 ± 1.46	81.91 ± 5.69	21	57.42 ± 2.90	23.78 ± 0.99	82.85 ± 3.64	5
7	Huahu, Zoige county	3448	33.92	102.87	46.10 ± 3.38	19.43 ± 1.59	69.12 ± 5.51	18	52.11 ± 4.20	21.10 ± 1.83	74.04 ± 5.11	7
8	Heihe'qiao, Zoige county	3453	33.60	102.93	50.68 ± 3.58	21.96 ± 1.11	78.45 ± 4.81	26	52.43 ± 3.01	22.02 ± 1.51	79.93 ± 7.57	10
9	Tangke, Zoige county	3450	33.58	102.90	50.40 ± 3.97	22.08 ± 1.36	78.71 ± 7.67	25	59.60 ± 5.98	23.81 ± 2.83	84.45 ± 9.29	6
10	Xiaman, Zoige county	3450	33.71	102.49	47.34 ± 3.12	20.19 ± 1.67	74.34 ± 6.22	5	49.54 ± 5.88	20.70 ± 2.42	72.06 ± 9.12	10

Table S2. Description of the morphological measurements.

Code	Measurement Description
SVL	Snout–vent length
LAHL	Length of lower arm and hand (from base of outer palmer tubercle to tip of third finger)
HILL	Length of hind limb (sum of thigh, tibia tarsus and foot length)

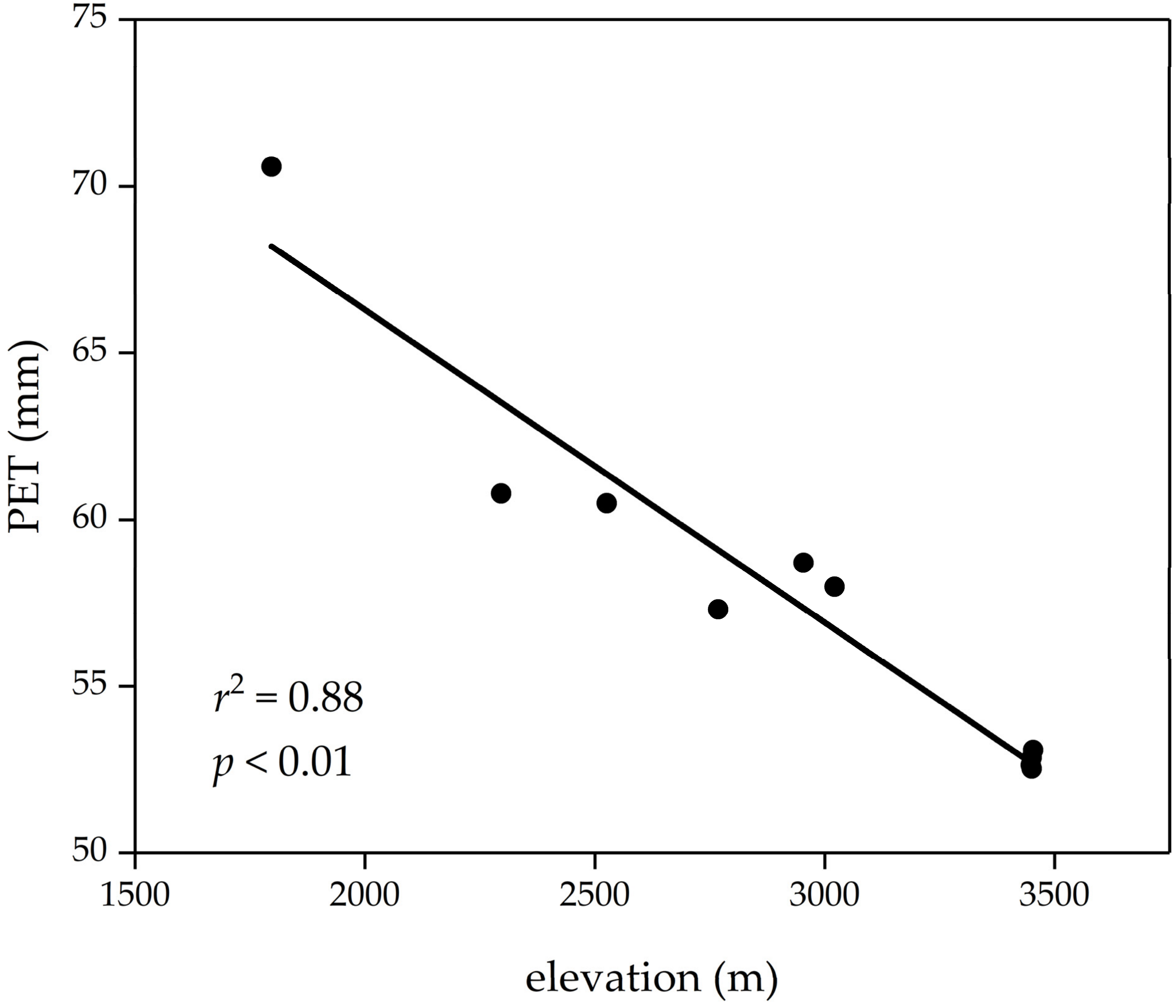

Figure S1. Variation of potential evapotranspiration (PET) along elevation on the eastern margin of the Qinghai-Tibetan Plateau.
